# Persistence of drug therapy is associated with ischemic stroke and other vascular events in high-risk stroke population

**DOI:** 10.3389/fneur.2022.925061

**Published:** 2022-07-25

**Authors:** Xingyang Yi, Hong Chen, Ming Yu, Hua Luo, Ju Zhou, Wei Wei, Yanfen Wang, Xiaorong Chen

**Affiliations:** ^1^Department of Neurology, People's Hospital of Deyang City, Deyang, China; ^2^Department of Neurology, Suining Central Hospital, Suining, China; ^3^Department of Neurology, The Affiliated Hospital of Southwest Medical University, Luzhou, China

**Keywords:** high risk stroke population, stroke, risk factors, health care, medication compliance, outcomes

## Abstract

The high-risk stroke populations are significantly associated with an increased risk of stroke or other vascular events. Although proven primary and secondary stroke prevention medications are available, persistent use is required to be effective. However, the persistence of drug therapy and its association with outcomes in the high-risk stroke population have received limited study in China. Hence, according to the China National Stroke Screening Survey (CNSSS) program in 2015, we performed this multicenter population-based cross-sectional survey and prospective cohort study in Sichuan of southwestern China. The residents aged ≥ 40 years volunteered to participate in a face-to-face survey in 8 communities. Subjects with at least three of eight stroke-related risk factors or a history of stroke were defined as high-risk stroke population. The interviewers recorded individuals' medications at a face-to-face survey, and all the high-risk stroke population was followed up for 4.7 years. The persistence of antihypertensives, hypoglycemics, lipid-lowering medications, and antithrombotics for stroke was evaluated. The primary outcome was new stroke. Secondary outcomes included new composite vascular events of stroke, myocardial infarction, and death during follow-up periods. Among 16,892 participants, 2,893 (17.1%) participants were high-risk stroke population and 2,698 (93.3%) participants completed to follow-up. The 4.7-year persistence of therapy rate of antihypertensives, hypoglycemics, lipid-lowering medications, and antithrombotics was 38.0%, 39.9%, 43.9%, and 59.8%, respectively. The total persistence of therapy rate for antihypertensives, hypoglycemics, lipid-lowering medications, and antithrombotics was 47.6% (136/286) in patients with hypertension, diabetes, dyslipidemia, and stroke at the same time. During the 4.7-year follow-up, there were 118 (4.4%) new ischemic stroke, 24 (0.9%) hemorrhagic stroke, 53 (2.0%) myocardial infarctions, and 33 (1.2%) deaths. After adjusting for the covariates, 4.7-year persistence of antihypertensives, hypoglycemics, lipid-lowering therapy, antithrombotics, and total persistence was independently associated with less new ischemic stroke and less new composite vascular events. Thus, more effective public education and efforts to understand and enhance the persistence of drug therapy are crucial to improve population health and decrease stroke and other vascular events for the high-risk stroke population.

## Introduction

Stroke is a leading cause of adult mortality and disability, and it has the greatest stroke burden in the world with a 39.3% risk of lifelong stroke for people over 25 years in China (1, 2). In the past few decades, the incidence of stroke has decreased because of good health services and effective prevention of risk factors for stroke in developed countries. However, the incidence of stroke has increased because of insufficiently good health services and strategies for primary prevention of stroke in developing countries (3). According to the report from the World Health Organization (WHO), the incidence of stroke is still increasing with an annual rate of 8.7% in China (4). There are approximately 3 million new stroke cases every year in China (1, 5).

In the last three decades, China has experienced rapid sociodemographic changes and health transitions, and the epidemiological features for stroke have likely changed in China (1, 5, 6). There was a large increase in the prevalence of hypertension, dyslipidemia, and diabetes mellitus (5–8), these were very common and modifiable risk factors for stroke, and all of these may affect stroke burden (1, 6). The incidence of stroke is significantly higher in the high-risk stroke population (multiple risk factors for stroke) than in those individuals with health or low-risk stroke population (1–3, 7); this indicates that the primary prevention and control of risk factors for stroke are very important to decrease the incidence of stroke.

Hypertension, dyslipidemia, diabetes mellitus, and history of stroke are the most important risk factors for stroke. Several medications, such as antihypertensives, lipid-lowering medications, hypoglycemics, and antithrombotics, had been demonstrated to reduce the risk of stroke among specific patient subgroups (9). However, the treatment rate, standard-reaching rate, and persistence of drug therapy rate for hypertension, diabetes mellitus, and dyslipidemia are significantly lower in China than in high-income countries (10, 11). Drugs don't work in patients who don't take them (12), and medications' non-persistence is very common and is associated with adverse outcomes in patients with coronary artery disease (13). Studies from secondary prevention for stroke have shown that implementation and persistence of secondary prevention medications after acute ischemic stroke are effective for preventing recurrent stroke (14, 15). In 2003, a WHO statement suggested that improved medication adherence “may have a far greater impact on the health of the population than any improvement in specific medical treatments” (13). Thus, effective control of risk factors requires more effective public education and greater responsibilities of individuals. These may increase the awareness of risk factors for stroke and the persistence of drug therapy (7, 11). However, the persistence of drug therapy and its association with outcomes in the high-risk stroke population have received limited study in China.

According to the China National Stroke Screening Survey (CNSSS) program (1), we performed a community-based high-risk stroke population survey in 8 communities in Sichuan of southwestern China (6). Using the data from the survey, we aimed to (1) investigate the persistence of drug therapy in high-risk population for stroke and (2) identify the association between persistence of drug therapy and outcomes during follow-up.

## Methods

### Study design and participants

This multicenter population-based cross-sectional survey and prospective cohort study was part of the CNSSS and was carried out in the Sichuan of southwestern China from May 2015 to January 2020. The study protocol was reviewed and approved by the Ethics Committee of participating hospitals (People's Hospital of Deyang City, the Affiliated Hospital of Southwest Medical University, and Suining Central Hospital), and informed consent was obtained from each participant during recruitment.

**Figure 1:**
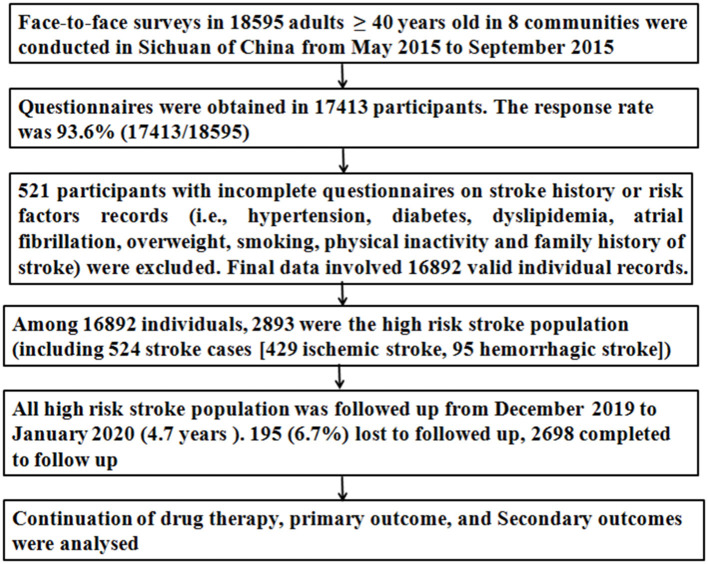
Flowchart in this study.

The cross-sectional survey was conducted in 8 communities of Sichuan from May 2015 to September 2015. The 8 communities were selected using the cluster randomization method. The detailed procedures for recruitment of participants and evaluation of risk factors are described in our previous article (6, 16) and Figure 1. In brief, all residents who aged ≥ 40 years and had lived in the county for at least 6 months were initially screened using a structured face-to-face questionnaire by interviewers. The questionnaire included demographic characteristics [e.g., age, gender, education level, employment, payment pattern of health insurance (urban basic medical insurance schemes, new rural cooperative medical schemes, and commercial insurance), and income], stroke-related behavioral factors (drinking, smoking, exercise habits, and diet), personal and family medical history of stroke, chronic diseases [hypertension, diabetes mellitus, dyslipidemia, and atrial fibrillation (AF)], current medications (antihypertensives hypoglycemics, lipid-lowering medications, and antithrombotics), and physical examination (height, weight, and resting blood pressure). Stroke history and stroke types (ischemic stroke and hemorrhagic stroke) were established by a combination of self-reporting and the judgment of a physician or neurologist according to neuroimaging. The eight risk factors for stroke, including overweight/obesity, smoking, physical inactivity, family history of stroke, hypertension, diabetes mellitus, dyslipidemia, and AF, were evaluated (6, 16). Subjects with at least three of the aforementioned eight stroke-related risk factors or a history of stroke were classified as high-risk population for stroke (6, 16, 17), otherwise, defined as low-risk population for stroke.

### Assessment of persistence of drug therapy

The interviewers asked the individuals who were taking medicines to continue medications at a face-to-face survey. The persistence of drug therapy was evaluated in the high-risk stroke population. In this study, we focused on several evidenced-based prevention medications including antihypertensives for hypertension (e.g., calcium-channel blockers, beta-blockers, angiotensin receptor blockers, angiotensin-converting enzyme inhibitors, and diuretics), hypoglycemics for diabetes mellitus (i.e., oral diabetic agents or insulin), antithrombotics for stroke (antiplatelet and anticoagulants), and lipid-lowering medications (statins or fibrates). Medication information at a face-to-face survey was used as the reference anchor for evaluating the persistence of drug therapy. To ensure proper persistence of drug therapy, trained research nurses were assigned to supervise the individuals' medication taking and advise the individuals to continue medications during follow-up periods once a year by telephone. The individuals in the high-risk stroke population were contacted from December 2019 to January 2020 (4.7 years after the face-to-face survey) using the telephone by trained research nurses or personnel.

The persistence of drug therapy was evaluated at 4.7 years after the face-to-face survey. In this study, 4.7-year persistence of drug therapy was defined as medication(s) continuation from a face-to-face survey to 4.7 years after the face-to-face survey (18, 19). Subjects who were prescribed an individual medication at a face-to-face survey but failed to take that medication at 4.7 years after the face-to-face survey or at every single point of telephone call each year were defined as “non-persistence.” The patients who had not received treatment at the survey but then received treatment during follow-up were also defined as “non-persistence.” However, subjects were considered “persistence,” if there was a switch of medication within the same class. The medication class included antihypertensives, hypoglycemics, antithrombotics, and lipid-lowering medications.

### Outcomes

All the high-risk stroke population was followed up from December 2019 to January 2020 by telephone interview and by reviewing the medical charts of each participant. The follow-up period was 4.7 years after the face-to-face survey. The primary outcome was new ischemic or hemorrhagic stroke during the follow-up. Stroke was defined as an acute focal neurological deficit that lasted for more than 24 h and was confirmed by brain computed tomography scan or magnetic resonance imaging scan. Secondary outcomes included new composite vascular events of stroke, myocardial infarction, or death from cardio-cerebral vascular cause during follow-up. Myocardial infarction was confirmed by the symptom of prolonged angina (>30 min), creatine kinase elevation, and electrocardiographic evidence of infarction (20). The researchers for outcome assessment were blinded to the status of persistence of drug therapy. For those individuals who reached at least one of the outcomes, a medical chart review was initiated to determine whether the event met the definitions described earlier.

### Statistical analysis

The sample size necessary for this study was calculated, and the detailed calculation for the sample size was described in our previous article (6). Finally, 18,595 participants aged ≥ 40 years participated in this survey (Figure 1).

Descriptive analyses were conducted to determine the distribution of the demographic data and risk factors in the study population using SPSS version 17.0 (SPSS Inc. New York, New York, United States). Categorical variables were presented as percentages and were compared using χ^2^ tests between groups. If continuous variables were normally distributed, they were expressed as mean ± standard deviation and were compared using the Student's *t-*test between groups. Otherwise, they were analyzed by the Wilcoxon rank-sum test. A multivariate logistic regression model was used to analyze the influence of the persistence of drug therapy on ischemic stroke and secondary outcomes. The variables that were statistically significant at *P* < 0.2 between patients with and without outcomes in the univariate analysis were entered into the multivariate logistic regression models. The results were reported as odds ratio (OR) with 95% confidential intervals (CIs).

All tests were two-sided, and *P* < 0.05 was considered statistically significant.

## Results

### The baseline characteristics of the high-risk stroke population

A total of 18,595 participants volunteered to participate in a face-to-face survey, questionnaires were obtained from 17,413 participants [the response rate was 93.6% (17,413/18,595)], and final data involved 16,892 valid individual records (Figure 1). Among 16,892 individuals, 2,893 (17.1%) were the high-risk stroke population. All the 2,893 high-risk stroke population was followed up at 4.7 years after a face-to-face survey, 2,698 completed to follow-up, and 195 (6.7%) lost to follow-up. The baseline characteristics of the 2,698 high-risk stroke population in a face-to-face survey are shown in Table 1. In the 2,698 high-risk stroke population, 1,949 (72.2%) had hypertension, 721 (26.7%) had diabetes mellitus, 751 (27.8%) had dyslipidemia, 487 (18.1%) had a history of stroke (399 were ischemic stroke and 88 were hemorrhagic stroke), and 378 had hypertension, diabetes, dyslipidemia, and stroke at the same time at a face-to-face survey.

**Table 1:** Baseline characteristics of the 2,698 high-risk stroke population at a face-to-face survey.

**Characteristics**	***N*** = **2,698**
Age ≥ 60 y (*n*, %)	1755 (65.0)
Male (*n*, %)	1,288 (47.7)
Rural (*n*, %)	1,456 (54.0)
Education (*n*, %)	
Junior middle school or below	2,345 (86.9)
Senior middle school or above	353 (13.1)
Overweight/obesity (*n*, %)	1,436 (53.2)
Smoking (*n*, %)	714 (26.5)
Physical inactivity (*n*, %)	1,647 (61.0)
Hypertension (*n*, %)	1,949 (72.2)
Diabetes (*n*, %)	721 (26.7)
Dyslipidemia (*n*, %)	751 (27.8)
Atrial fibrillation (*n*, %)	71 (2.6)
Family history for stroke (*n*, %)	479 (17.8)
History of ischemic stroke (*n*, %)	399 (14.8)
History of hemorrhagic stroke (*n*, %)	88 (3.3)
Hypertension, diabetes, dyslipidemia and stroke at same time	378 (14.0)

### Persistence of drug therapy in the high-risk stroke population

At a face-to-face survey, 996 (51.1%) patients with hypertension received antihypertensive treatment, 444 (61.6%) patients with diabetes mellitus received hypoglycemics, and 374 (49.8%) patients with dyslipidemia received lipid-lowering therapy; however, all patients with a history of stroke received antithrombotics. Among the 378 participants with hypertension, diabetes, dyslipidemia, and stroke at the same time, 286 (75.7%) received antihypertensives, hypoglycemic, lipid-lowering therapy, and antithrombotics at the same time. At 4.7 years after the survey, only 378 (19.4%) patients with hypertension continued taking antihypertensives, 177 (24.5%) patients with diabetes mellitus continued taking hypoglycemics, 164 (21.8%) patients with dyslipidemia continued taking lipid-lowering medications, 291 (75.2%) patients with a history of stroke continued taking antithrombotics, and 136 (36.%) patients with hypertension, diabetes, dyslipidemia, and stroke continued taking antihypertensives, hypoglycemics, lipid-lowering medications, and antithrombotics at the same time prescribed at a face-to-face survey. The persistence of therapy rate of antihypertensives, hypoglycemics, lipid-lowering medications, and antithrombotics was 38.0% (378/996), 39.9% (177/444), 43.9% (164/374), and 59.8% (291/478), respectively (Table 2). The total persistence of therapy rate for antihypertensives, hypoglycemics, lipid-lowering medications, and antithrombotics was 47.6% (136/286) in patients with hypertension, diabetes, dyslipidemia, and stroke at the same time (Table 2).

**Table 2:** Persistence of medications in the 2,698 high-risk stroke population.

	**Hpertension (*****n*** = **1,949)**	**Diabetes** **(*****n*** = **721)**	**Dyslipidemia (*****n*** = **751)**	**History of stroke** **(*****n*** = **487)**	**Hpertension, diabetes, dyslipidemia and stroke at same time (*****n*** = **378)**
Treatment at survey (*n*, %)	996 (51.1)	444 (61.6)	374 (46.2)	487 (100.0)	286 (75.7)
Treatment at 4.7 years after survey (*n*, %)	378 (19.4)	177 (24.5)	164(21.8)	291(59.8)	136 (36.0)
Persistence of therapy rate (*n*, %)	38.0 (378/996)	39.9 (177/444)	43.9 (164/374)	59.8 (291/487)	47.6 (136/286)

### Outcomes and their association with persistence of drug therapy

In a total of the 2,893 high-risk stroke population, 2,698 (93.3%) completed a 4.7-year follow-up, and there were 118 (4.4%) new ischemic stroke, 24 (0.9%) hemorrhagic stroke, 53 (2.0%) myocardial infarctions, and 33 (1.2%) deaths. Compared with the patients without outcomes, the patients with outcomes were older and had a higher history of stroke and a lower rate of antihypertensive therapy (*P* < 0.05, Table 3). However, there was no significant difference in other risk factors between patients with and without outcomes (*P* > 0.05, Table 3).

**Table 3:** Comparison of patients with and without outcomes during follow-up periods.

**Characteristics**	**Patients with outcomes (*****n*** = **192)**	**Patients without outcomes (*****n*** = **2,506)**	***P*** **value**
Age ≥60y (*n*, %)	138 (71.9)	1,617 (64.5)	0.041
Male (*n*, %)	89 (46.3)	1,199 (47.8)	0.705
Rural (*n*, %)	104 (54.2)	1,352 (54.0)	0.983
Education (*n*, %)			
Junior middle school or below	169 (88.0)	2,176 (86.8)	0.664
Senior middle school or above	23 (12.0)	330 (13.2)	
Overweight/obesity (*n*, %)	107 (55.7)	1,329 (53.0)	0.512
Smoking (*n*, %)	57 (29.7)	657 (26.2)	0.294
Physical inactivity (*n*, %)	114 (59.4)	1,533 (61.2)	0.641
Hypertension (*n*, %)	136 (70.8)	1,813 (72.3)	0.672
Antihypertensive drugs (*n*, %)	52 (27.1)	944 (37.7)	0.003
Diabetes (*n*, %)	59 (30.7)	662 (26.4)	0.203
Hypoglycemic drugs (*n*, %)	37 (19.3)	407 (16.2)	0.986
Dyslipidemia (*n*, %)	57 (29.7)	694 (27.7)	0.571
Lipid lowering therapy (*n*, %)	23 (12.0)	351 (14.0)	0.148
Atrial fibrillation (*n*, %)	4 (2.1)	67 (2.7)	0.641
Family history for stroke (*n*, %)	40(20.8)	439 (17.5)	0.246
History of stroke (*n*, %)	82(42.7)	405(16.2)	<0.001

The association between 4.7-year persistence of drug therapy and clinical outcomes is shown in Table 4. Compared with patients with 4.7-year non-persistence, patients with 4.7-year persistence of antihypertensives had a significantly lower rate of new ischemic stroke (persistence vs. non-persistence: 1.3% vs. 4.2%, *P* = 0.013) and total new composite vascular events (persistence vs. non-persistence: 3.2% vs. 8.4%, *P* = 0.001). Compared with patients with 4.7-year non-persistence of lipid-lowering medications, patients with 4.7-year persistence had a significantly lower rate of total new composite vascular events (persistence vs. non-persistence: 3.7% vs. 10.0%, *P* = 0.017). Compared with patients with 4.7-year non-persistence of antithrombotics, patients with 4.7-year persistence had a significantly lower rate of new ischemic stroke (persistence vs. non-persistence: 5.8% vs. 15.8%, *P* < 0.001) and total new composite vascular events (persistence vs. non-persistence: 14.8% vs. 27.0%, *P* < 0.001). Furthermore, the total persistence of antihypertensives, hypoglycemics, lipid-lowering medications, and antithrombotics in patients with hypertension, diabetes, dyslipidemia, and stroke at the same time was significantly associated with a lower rate of new ischemic stroke (persistence vs. non-persistence: 0.74% vs. 6.7%, *P* = 0.009) and total new composite vascular events (persistence vs. non-persistence: 2.2% vs. 12.7%, *P* < 0.001). However, there was no significant difference in outcomes between patients with compliance and non–compliance of hypoglycemics by univariate analysis (*P* > 0.05, Table 4).

**Table 4:** Persistence of drug therapy with outcomes (*n*, %).

	**Ischemic stroke**	**Hemorrhagic stroke**	**Myocardial infarction**	**Death**	**Total**
**Hypertension**					
No (*n* = 749)	32 (4.3)	7 (0.9)	19 (2.5)	6 (0.8)	64 (8.5)
Yes (*n* = 1,949)	86 (4.4)	17 (0.9)	34 (1.74)	27 (1.4)	164 (8.4)
*P* value	0.943	0.951	0.197	0.211	0.910
Non-treatment (*n* = 953)	55 (5.8)	11 (1.2)	20 (2.1)	14 (1.5)	100 (10.5)
Treatment (*n* = 996)	31 (3.1)	6 (0.6)	14 (1.4)	13 (1.3)	64 (6.6)
*P* value	0.004	0.190	0.243	0.757	0.002
Non-persistence (*n* = 618)	26 (4.2)	5 (0.8)	11 (1.8)	10 (1.6)	52 (8.4)
Persistence (*n* = 378)	5 (1.3)	1 (0.3)	3 (0.8)	3(0.8)	12 (3.2)
*P* value	0.013	0.417	0.271	0.390	0.001
**Diabetes**					
No (*n* = 1,977)	81 (4.1)	18 (0.9)	37 (1.9)	22 (1.1)	158 (8.0)
Yes (*n* = 721)	37 (5.1)	6 (0.8)	16 (2.2)	11 (1.5)	70 (9.7)
*P* value	0.707	0.976	0.573	0.397	0.167
Non-treatment (*n* = 277)	15 (5.4)	3 (1.1)	5 (1.8)	4 (1.4)	27 (9.7)
Treatment (*n* = 444)	22 (5.0)	3 (0.7)	11 (2.5)	7 (1.6)	43 (9.7)
*P* value	0.785	0.680	0.614	0.100	0.999
Non-persistence (*n* = 267)	17 (6.4)	2 (0.7)	7 (2.6)	5 (1.9)	31 (11.6)
Persistence (*n* =1 77)	5 (2.8)	1 (0.6)	4 (2.3)	2 (1.1)	12 (6.8)
*P* value	0.118	0.100	0.100	0.708	0.091
**Dyslipidemia**					
No (*n* = 1,947)	81 (4.2)	18 (0.9)	36 (1.8)	23 (1.2)	158 (8.1)
Yes (*n* = 751)	37 (4.9)	6 (0.8)	17 (2.3)	10 (1.3)	70 (9.2)
*P* value	0.394	0.768	0.495	0.722	0.304
Non-treatment (*n* = 377)	23 (6.1)	4 (1.1)	11 (2.9)	5 (1.3)	43 (11.4)
Treatment (*n* = 374)	14 (3.7)	2 (0.5)	6 (1.6)	5 (1.3)	27 (7.2)
*P* value	0.136	0.686	0.226	0.100	0.046
Non-persistence (*n* = 210)	11 (5.2)	2 (1.0)	5(2.4)	3(1.4)	21(10.0)
Persistence (*n* = 164)	3 (1.8)	0 (0.0)	1(0.6)	2(1.2)	6(3.7)
*P* value	0.102	0.505	0.234	0.100	0.017
**Antithrombotics**					
Non-persistence (*n* = 196)	31 (15.8)	3 (1.5)	12 (6.1)	7 (3.6)	53 (27.0)
Persistence (*n* = 291)	17 (5.8)	8 (2.7)	11 (3.8)	7 (2.4)	43 (14.8)
*P* value	<0.001	0.378	0.211	0.473	<0.001
**Total persistence for antihypertensives, hypoglycemics, lipid lowering medications and antithrombotics at same time**					
Yes (*n* = 136)	1 (0.74)	0 (0.0)	1 (0.74)	1 (0.74)	3 (2.2)
No (*n* = 150)	10 (6.7)	2 (1.3)	4 (2.7)	3 (2.0)	19 (12.7)
*P* value	0.009	0.192	0.223	0.391	<0.001

### Effect of persistence of drug therapy on outcomes by multivariable regression analysis

The effect of 4.7-year persistence of drug therapy on new ischemic stroke and new total composite vascular events was evaluated using multivariable regression analysis. Variables entered the models were the variables showed a significant association (*P* < 0.2) with new ischemic stroke and total new composite vascular events on univariate analysis. After adjusting for these covariates, 4.7-year persistence of antihypertensives, hypoglycemics, lipid-lowering therapy, antithrombotics, and total persistence was significantly independently associated with less new ischemic stroke (OR, 0.81, 95% CI: 0.66–0.94, *P* < 0.001; OR, 0.92, 95% CI: 0.76–0.97, *P* = 0.029; OR, 0.90, 95% CI: 0.82–0.95, *P* = 0.029; OR, 0.69, 95% CI: 0.64–0.89, *P* < 0.001; OR, 0.69, 95% CI: 0.62–0.87, *P* < 0.001, respectively, Table 5) and less total new composite vascular events (OR, 0.76, 95% CI: 0.63–0.92, *P* < 0.001; OR, 0.92, 95% CI: 0.75–0.97, *P* = 0.027; OR, 0.85, 95% CI: 0.77–0.95, *P* = 0.015; OR, 0.73, 95% CI: 0.62–0.92, *P* < 0.001; OR, 0.70, 95% CI: 0.61–0.89, *P* < 0.001, respectively, Table 6).

**Table 5:** Multivariable regression analysis of risk factors for new ischemic stroke during follow-up.

**Factor**	**OR**	**95% CI**	***P*** **value**
Age ≥ (60 years)	1.04	0.77–1.62	0.235
Antihypertensives for hypertension	0.94	0.88–1.21	0.362
Persistence of antihypertensives	0.81	0.66–0.94	<0.001
Persistence of hypoglycemic	0.92	0.76–0.97	0.029
Lipid lowering therapy for dyslipidemia	0.95	0.93–1.19	0.422
Persistence of lipid lowering therapy	0.90	0.82–0.95	0.029
History of stroke	2.23	1.37–5.18	<0.001
Persistence of antithrombotics for stroke	0.69	0.64–0.89	<0.001
Total persistence for antihypertensives, hypoglycemics, lipid lowering medications and antithrombotics	0.68	0.62–0.87	<0.001

OR, odds ratio; CI, confidence interval.

**Table 6:** Multivariable regression analysis of risk factors for total new composite vascular events during follow-up.

**Factor**	**OR**	**95% CI**	***P*** **value**
Age ≥ (60 years)	1.13	0.94–2.56	0.118
Antihypertensives for hypertension	0.91	0.86–1.24	0.257
Persistence of antihypertensives	0.76	0.63–0.92	<0.001
Diabetes	1.12	0.92–3.13	0.214
Persistence of hypoglycemic	0.92	0.75–0.97	0.027
Lipid lowering therapy for dyslipidemia	0.95	0.89–1.08	0.214
Persistence of lipid lowering therapy	0.85	0.77–0.95	0.015
History of stroke	2.52	1.61–6.06	<0.001
Persistence of antithrombotics for stroke	0.73	0.62–0.92	<0.001
Total persistence for antihypertensives, hypoglycemics, lipid lowering medications and antithrombotics	0.70	0.61–0.89	<0.001

OR, odds ratio; CI, confidence interval.

## Discussion

In this study, using our data from a community-based study in Sichuan of southwestern China (6, 16, 21), we found that the treatment rate for hypertension, diabetes mellitus, and dyslipidemia was very low, and the persistence of antihypertensives, hypoglycemics, lipid-lowering drugs, and antithrombotics was significantly associated with a decreased risk of new ischemic stroke and total new composite vascular events in the high-risk stroke population.

Hypertension, diabetes mellitus, dyslipidemia, and stroke are the most important risk factors for stroke or stroke recurrence in China (1, 6, 7). Antihypertensives, hypoglycemics, lipid-lowering therapy, and antithrombotics (for stroke) had been demonstrated to reduce the risk of stroke or other vascular events (9). However, the treatment rate and standard-reaching rate for hypertension, diabetes mellitus, and dyslipidemia are significantly lower in China than in high-income countries (10, 11). The proportion of people whose hypertension is controlled is under 20% in China; similarly, dyslipidemia and diabetes are poorly controlled in China (10, 11, 22). In this survey, we found that the proportion of treatment for the patients with hypertension, diabetes mellitus, and dyslipidemia was 51.1%, 61.6%, and 46.2% in a face-to-face survey, respectively, which was considerably lower than in the United Kingdom or the United States (23). Thus, improved control of these factors requires more effective public education and greater responsibilities of individuals in China.

“Drugs don't work in patients who don't take them” (13). The persistence of drug therapy is critical and is a common and refractory problem. Medication non-persistence is very common and is associated with adverse outcomes and higher costs of care in the world (12, 13, 24). Improved medication adherence may have a far greater impact on the health of the population than any improvement in specific medical treatments (12). Previous studies on the persistence of secondary prevention medications after acute ischemic stroke or transient ischemic attack (TIA) in the Chinese population have been reported (15, 25). The study from the China National Stroke Registry (CNSR) showed that only 63.6% of patients with acute ischemic stroke or TIA continued taking all the secondary prevention medications prescribed at hospital discharge for 3 months after discharge. By medication class, 3-month compliance was found highest for diabetic medications (82.7%), followed by antiplatelet agents (80.4%) and antihypertensives (79.2%), and lowest for warfarin (31.7%) (15). Another study from China found that antihypertensive use was well-maintained, whereas the compliance rate of antiplatelet was 66% at 12 months after stroke (25). These studies from China were in accordance with other international studies (26, 27). The adherence evaluation after ischemic stroke longitudinal (AVAIL) study showed that nearly one-quarter and one-third of patients with acute ischemic stroke discontinued one or more of their prescribed secondary prevention medications at 3- and 12-months postdischarge, respectively (26). An inner-city population study reported that the adherence rate was 70% for antihypertensive therapy, 75% for antiplatelet therapy, and 41% for anticoagulation therapy for 3 months after ischemic stroke (27). In this study, we found that a 4.7-year compliance rate for antithrombotics was not ideal (59.8%) in patients with stroke, and it was significantly lower compared with other previous studies (15, 25–27). The reason may be related to the long follow-up time of this study (4.7 years). To the best of our knowledge, this study is the longest follow-up to identify compliance for antithrombotics after stroke.

Till present, few studies investigated the persistence of drug therapy and its association with outcomes in the high-risk stroke population. In this study, the results showed that the 4.7-year persistence of therapy rate for antihypertensives, hypoglycemics, and lipid-lowering medications was 38.0%, 39.9%, and 43.9%, respectively, indicating the persistence of drug therapy is very low in the high-risk stroke population in China. A study from Systolic Blood Pressure Intervention Trial (SPRINT) data reported that 21.2% had low antihypertensives adherence, 40.0% had medium adherence, and 38.8% had high adherence for patients with hypertension, and medium or above compliance was significantly associated with lower systolic blood pressure (SBP) (28), and this was agreed with this study.

Numerous studies have shown that the persistence of secondary prevention medications, including antiplatelet, warfarin, statins, antihypertensive, and antidiabetic medications was associated with a lower hazard of recurrent stroke, composite events, death, and lower OR of disability in patients with ischemic stroke or TIA (15, 29, 30). In this study, we found that the 4.7-year persistence of antithrombotics in patients with stroke was independently associated with less recurrent ischemic or hemorrhagic stroke and less total new composite vascular events, and the results were agreed with other previous studies (15, 29, 30). There are few studies to evaluate the association between persistence of drug therapy and outcomes in the high-risk stroke population. Studies from patients with coronary artery disease have shown that medication non-persistence for coronary artery disease is associated with poor blood pressure control and subsequent adverse outcomes (12, 13). Studies from SPRINT data showed that medium or above compliance with antihypertensives was significantly associated with lower SBP (28) but was not associated with primary outcomes (a composite of myocardial infarction, other acute coronary syndromes, heart failure, stroke, or death from cardiovascular causes) (31). In this study, the results showed that the 4.7-year persistence of antihypertensives, hypoglycemics, and lipid-lowering therapy was significantly associated with less new ischemic stroke and total new composite vascular events in the high-risk stroke population. Thus, more effective public education, greater responsibilities of individuals, and efforts to understand and enhance the persistence of drug therapy are crucial to improve population health and decrease stroke or other vascular events for the high-risk stroke population (7), and fixed-dose combination options for polypill products would be more eligible for secondary and primary prevention of stroke (32).

There are several limitations in our study. First, because numerous previous studies have evaluated the influence factors of persistence of drug therapy, the main aim of this study was to investigate the association between persistence of drug therapy and outcomes in the high-risk stroke population. Thus, we did not assess the influence factors of persistence of drug therapy in this study. Second, this study was a multicenter, cross-sectional survey, and prospective cohort study in southwestern China, and there may have a recall bias because of the self-reported questionnaire and follow-up by telephone. Third, we only screened residents aged ≥ 40 years in 8 communities in southwestern China. Thus, the results of this study may not represent the full spectrum of the Chinese population. The findings must be validated in larger, multicenter studies in China. Fourth, some studies showed that the standard-reaching rate for hypertension, diabetes mellitus, and dyslipidemia (embodied in blood pressure, glucose, and lipids) was very low, and was associated with outcomes (10, 11). However, some studies thought that blood pressure, blood glucose, and lipids, as the intermediate markers during medication treatment, were not associated with susceptibility to stroke and outcomes (33, 34). In this study, although we investigated the persistence of antihypertensives, hypoglycemics, lipid-lowering medications, and antithrombotics in the high-risk stroke population, the relevant data such as blood pressure, glucose, and lipids at 4.7 years after a face-to-face survey were not collected. Thus, future studies are needed to evaluate the effect of blood pressure, blood glucose, and lipids on outcomes. Furthermore, as the prevalence of cardiogenic stroke was very low in the survey, we did not stratify the compliance with anticoagulation therapy and its effect on outcomes. Finally, our measure of the persistence of drug therapy was telephone self-reported compliance. Although the excellent agreement between telephone self-reported compliance and analysis of pharmaceutical data has been previously reported (35), such data might be biased by patients' subjective response (information bias and recall bias). The 8-item Morisky Medication Adherence Scale (MMAS-8) is an 8-question self-reported instrument that has proven to be a valid and reliable assessment tool for compliance (28, 31). Further studies are needed to determine the association between medication compliance and outcomes using MMAS-8 in the high-risk stroke population.

## Conclusion

In this study, we found that the 4.7-year persistence of antihypertensives for hypertension, hypoglycemics for diabetes, lipid-lowering medications for dyslipidemia, and antithrombotics for stroke was very low, and the persistence of antihypertensives, hypoglycemics, lipid-lowering medications, and antithrombotics was independently associated with less new ischemic stroke and total new composite vascular events in the high-risk stroke population. Thus, more effective public education and efforts to understand and enhance the persistence of drug therapy are crucial to improve population health and decrease stroke and other vascular events for the high-risk stroke population.

## Data availability statement

The raw data supporting the conclusions of this article will be made available by the authors, without undue reservation.

## Ethics statement

The studies involving human participants were reviewed and approved by Ethics Committee of People's Hospital of Deyang City. The patients/participants provided their written informed consent to participate in this study.

## Author contributions

XY and HC: designed the study and acquired funding. MY, HL, and JZ: performed this survey and follow-up. WW, YW, and XC: analyzed the results and drafted the figures. XY, MY, and HL: drafted manuscript and the tables. XY, HC, MY, and HL: supervised the project. All authors contributed to the article and approved the submitted version.

## Funding

This study was supported in part by grants from the Scientific Research Foundation of Sichuan Provincial Health Department (Grant No. 16ZD046). The funding body did not participate in the design of the study; collection, analysis, and interpretation of data; and in writing the manuscript.

## Conflict of interest

The authors declare that the research was conducted in the absence of any commercial or financial relationships that could be construed as a potential conflict of interest.

## Publisher's note

All claims expressed in this article are solely those of the authors and do not necessarily represent those of their affiliated organizations, or those of the publisher, the editors and the reviewers. Any product that may be evaluated in this article, or claim that may be made by its manufacturer, is not guaranteed or endorsed by the publisher.
